# Tuberculin Skin Test Reversion following Isoniazid Preventive Therapy Reflects Diversity of Immune Response to Primary *Mycobacterium tuberculosis* Infection

**DOI:** 10.1371/journal.pone.0096613

**Published:** 2014-05-05

**Authors:** Denise F. Johnson, LaShaunda L. Malone, Sarah Zalwango, Joy Mukisa Oketcho, Keith A. Chervenak, Bonnie Thiel, Harriet Mayanja-Kizza, Catherine M. Stein, W. Henry Boom, Christina L. Lancioni, for the Tuberculosis Research Unit

**Affiliations:** 1 Tuberculosis Research Unit (TBRU), Case Western Reserve University, Cleveland, Ohio, United States of America; 2 Uganda-Case Western Reserve University Research Collaboration, Kampala, Uganda; 3 Department of Medicine, Makerere University College of Health Science, Kampala, Uganda; 4 Department of Epidemiology and Biostatistics, Case Western Reserve University, Cleveland, Ohio, United States of America; 5 Department of Pediatrics, Oregon Health & Science University, Portland, Oregon, United States of America; University of Cape Town, South Africa

## Abstract

**Rationale:**

Healthy household contacts (HHC) of individuals with Tuberculosis (TB) with Tuberculin Skin Test (TST) conversions are considered to harbor latent *Mycobacterium tuberculosis* (Mtb), and at risk for TB. The immunologic, clinical, and public health implications of TST reversions that occur following Isoniazid preventive therapy (IPT) remain controversial.

**Objectives:**

To measure frequency of TST reversion following IPT, and variation in interferon-gamma (IFN-γ) responses to Mtb, in healthy Ugandan TB HHC with primary Mtb infection evidenced by TST conversion.

**Methods:**

Prospective cohort study of healthy, HIV-uninfected, TST-negative TB HHC with TST conversions. Repeat TST was performed 12 months following conversion (3 months following completion of 9 month IPT course) to assess for stable conversion *vs.* reversion. Whole blood IFN-γ responses to Mtb antigen 85B (MtbA85B) and whole Mtb bacilli (wMtb) were measured in a subset (n = 27 and n = 42, respectively) at enrollment and TST conversion, prior to initiation of IPT.

**Results:**

Of 122 subjects, TST reversion was noted in 25 (20.5%). There were no significant differences in demographic, clinical, or exposure variables between reverters and stable converters. At conversion, reverters had significantly smaller TST compared to stable converters (13.7 mm vs 16.4 mm, respectively; p = 0.003). At enrollment, there were no significant differences in IFN-γ responses to MtbA85B or wMTB between groups. At conversion, stable converters demonstrated significant increases in IFN-γ responses to Ag85B and wMtb compared to enrollment (p = 0.001, p<0.001, respectively), while there were no significant changes among reverters.

**Conclusions:**

TST reversion following IPT is common following primary Mtb infection and associated with unique patterns of Mtb-induced IFN-γ production. We have demonstrated that immune responses to primary Mtb infection are heterogeneous, and submit that prospective longitudinal studies of cell mediated immune responses to Mtb infection be prioritized to identify immune phenotypes protective against development of TB disease.

## Introduction

Although the incidence of Tuberculosis disease (TB) caused by *Mycobacterium tuberculosis* (Mtb) has been declining, Mtb continues to contribute dramatically to human morbidity and mortality worldwide with 8.3 million new cases estimated in 2012, and 1.3 million deaths due to TB [1]. Limitations in our understanding of the natural history of Mtb infection have led to the presumption that all otherwise healthy Mtb-exposed individuals with evidence for Mtb-sensitization [demonstrated by a positive Tuberculin skin test (TST) or Mtb-specific interferon-gamma release assay (IGRA)] harbor a latent form of Mtb and thus remain at life-long risk for Tuberculosis disease (TB) [2]. However, as only a small proportion (5–10%) of healthy Mtb-sensitized individuals develop TB in the absence of anti-mycobacterial prophylaxis [3], clearance of Mtb-infection, rather than just successful containment of the bacilli, may be feasible. Broadening our understanding of potential outcomes following Mtb infection is required for development of refined bioassays that quantify an individual's Mtb bacillary load following exposure, predict disease progression, and to identify markers of protective immunity that can be incorporated into TB vaccine trials.

The biology of human primary Mtb-infection is difficult to study as the majority of sensitized individuals are either uncertain when their exposure occurred, have experienced multiple lifetime or on-going TB exposure, or have been exposed to non-tuberculosis mycobacteria (NTM) and/or the Bacillus Calmette-Guérin (BCG) vaccine that compromise TST specificity [4]. These limitations complicate the interpretation of early studies of Mtb infection that relied on TST as sole marker of Mtb-sensitization [5]–[9], and in some circumstances have impeded modern studies utilizing serial IGRAs [10]–[12]. We hypothesized that following Mtb-exposure a subset of individuals who have experienced primary Mtb infection will successfully eliminate their infection, and that elimination of Mtb will be reflected by changes in immunological measures of Mtb-sensitization. To explore this hypothesis we monitored participants of a well-established, longitudinal study of Mtb household contacts (HHC) in Uganda [13], [14] who developed evidence of primary Mtb infection following enrollment, with a combination of serial TSTs and an in-house, Mtb-specific whole blood IGRA.

## Patients, Materials, and Methods

### Ethics Statement

Ethics approval for this study was provided by the AIDS Scientific Committee of Makerere University, the Uganda National Council on Science and Technology, and the institutional review board at University Hospitals Case Medical Center, Cleveland, OH. Written informed consent was obtained from all participants in their local language, with parents/guardians providing written consent for children under 18 years old.

### Patients

Between April 2002-December 2010, adults with active, culture confirmed pulmonary TB and their HHC of all ages were enrolled and followed for up to 24 months in a prospective cohort study of Mtb infection and disease in the Kawempe Division of Kampala, Uganda, as has been previously described [13]–[16]. HHC were defined as individuals who had lived in the same home as the TB index case for a minimum of 7 consecutive days during the previous 3 months. For this sub-study of primary Mtb-infection, only HHC who were asymptomatic, HIV-uninfected, and TST-negative at enrollment with a documented subsequent TST conversion were included. Additional inclusion criteria included acceptance of isoniazid (INH) preventive therapy (IPT) following TST conversion, and having a documented repeat TST placed 12 months following initial conversion. HHC with a history of prior TB disease were excluded. Compliance with IPT was not formally assessed in this current study. However, analysis of IPT compliance performed from April 2002-December 2006 among TB HHC from the same parent study, demonstrated 86% adherence in 361 HHC using reported number of doses consumed. 60% adherence with IPT was identified among 190 HHC using INH urine metabolites as evidence of compliance [17].

### Study Investigations & Definitions

HHC were evaluated at enrollment with a standardized questionnaire about TB risk factors and symptoms, physical exam (PE), chest x-ray (CXR), and HIV serology or DNA PCR for children <18 months old. Blood for our in-house, whole blood IGRA was drawn at enrollment, followed by placement of TST using Mantoux method (0.1 mL of five tuberculin units of purified protein derivative, Tubersol; Cannaught Laboratories Limited, Willowdale, Ontario, Canada). TST was placed on the volar surface of the forearm and read after 48–72h by experienced home study visitors as the diameter (mm) of palpable induration [15]. TST was repeated at 3, 6, 12, 18, and 24 months. TST conversion was defined for individuals >5 years as: 1) enrollment TST <10 mm; 2) TST induration of ≥10 mm upon repeat testing; and 3) an increase in TST induration of ≥6 mm upon repeat testing. For children <5 years TST conversion was defined as: 1) enrollment TST <5 mm; 2) TST induration of ≥5 mm upon repeat testing; and 3) an increase in TST induration of ≥3 mm upon repeat testing. A 9 month course of INH preventive therapy was prescribed to all TST converters once TB disease was ruled-out [18]. A repeat TST was placed 12 months following initial conversion to assess for reversion versus a persistently positive TST (stable conversion). TST reversion was defined for individuals >5 years as repeat TST ≤10 mm with ≥6 mm decrease from conversion; for children <5 years reversion was defined as repeat TST ≤5 mm with ≥3 mm decrease from conversion [19], [20]. Due to the risk of TB disease among young HHC [21], IPT was offered to all HHC <5 years at enrollment once TB disease was ruled-out. Three children in the reverter group and 6 children in the stable converter group received 30+ days of INH prior to initial TST conversion (p = 0.40).

### Whole blood interferon-gamma release assay (WB-IGRA)

Whole blood (1 ml/kg, maximum 10 ml) was collected at study enrollment and at TST conversion, and diluted 1∶5 with supplemented RPMI-1640 medium (Lonza, Walkersville, MD). 1 ml of diluted whole blood was cultured at 37° in 5% CO_2_ in 24-well tissue cultures plates with: whole Mtb H37Ra (10^6 ^CFU/ml), and Mtb Ag85B (10 µg/ml; Colorado State University). Whole blood incubated with phytohaemagglutinin (PHA; 5 µg/ml; Sigma) and medium alone served as positive and negative controls, respectively. Culture supernatants were collected after 7 days and stored at –80°C until batch tested for IFN-γ using a commercial enzyme-linked immunosorbent assay (ELISA; range 25.6–1000 pg/ml; Endogen, Woburn, MA). Reported results reflect stimulated values minus medium alone (unstimulated controls) values, with corrections made for initial whole blood dilution. Values above the upper-limit of detection of the assay were set at 5000 pg/ml. WB-IGRA was performed in a subset of eligible HHC at enrollment and time of initial TST conversion. Although blood draw for WB-IGRA was attempted for all participants at both time points, in some cases blood draws were missed, delayed, or declined by participants or their guardians. In total, WB-IGRA results for Ag85B and wMTB were available for 27 and 42 participants, respectively. Fewer subjects had data available for Ag85B WB-IGRA responses as this antigen was introduced into the study protocol late into the 8 year study time frame.

### Statistical Analysis

Univariate analysis was performed to identify demographic and clinical differences between stable converters and individuals with TST reversions. For continuous variables, Student's T test was applied to data with normal distribution and Wilcoxon Scores (rank sums) were computed using the Wilcoxon Two-sample Test for non-normally distributed data. Categorical variables were tested using the chi-square test. A p-value of <0.05 was considered significant. To evaluate whether epidemiological risk factors significantly differed for reverters versus stable converters, we modified the risk score of Mandalakas, *et al* (Table S1) [22]. Mandalakas, *et al.*, developed a 10-point exposure score for child HHC of TB cases. Our epidemiologic study collected all but one of these variables (“Is the index case the primary caregiver of the child?”). Thus in children age <15, our modified risk score ranged up to 9. We further modified this risk score for adults age ≥15 in two ways: 1) Instead of including whether the index case was the mother of the child, we included whether the adult was the spouse of the index case; 2) Instead of including whether the index case was the primary caregiver of the child, we included whether the HHC was the primary caregiver of the index case. Thus, this variable is on a 10-point scale in adults. SAS version 9.3 software (SAS Institute Inc, Cary, NC, USA) was used for all analysis.

## Results

### Identification and characteristics of subjects with stable TST conversions and TST reversions

There were a total of 122 healthy, HHC who were TST negative at study enrollment and met criteria for inclusion in this analysis. We identified 97 individuals with stable TST conversions and 25 individuals with TST reversions, yielding a 20.5% prevalence of TST reversion in our population. The demographic and clinical characteristics of all subjects are shown in Table 1. Notably, there were no significant differences in age, gender, presence of BCG scar, or Body Mass Index (BMI) between stable converters and reverters. No subjects in either group received treatment for TB disease during the course of the study.

**Table 1:** Demographic and clinical characteristics of HHC with TST conversion.

	Totaln = 122	Stable Converters n = 97 (79.5%)	Revertersn = 25 (20.5%)	P*-*value
Gender				
Male	45	33 (34%)	12 (48%)	0.20
Female	77	64 (66%)	13 (52%)	
Age (median)	122	13.0	7.0	0.07
Age group				
< 5 years	24	15 (15%)	9 (36%)	0.37
5–15 years	55	46 (47%)	9 (36%)	
16–25 years	32	26 (27%)	6 (24%)	
26–45 years	8	7 (7%)	1 (4%)	
> 45 years	3	3 (3%)	0	
BCG scar present				
Yes	82	67 (69%)	15 (60%)	0.44
No	25	18 (19%)	7 (28%)	
Uncertain	15	12 (12%)	3 (12%)	
BMI (median)	122	18.2	16.8	0.44
Treated for TB Disease				
Yes	0	0	0	-
No	122	97 (100%)	25 (100%)	

### Serial TST measurements among study participants

**Figure 1:**
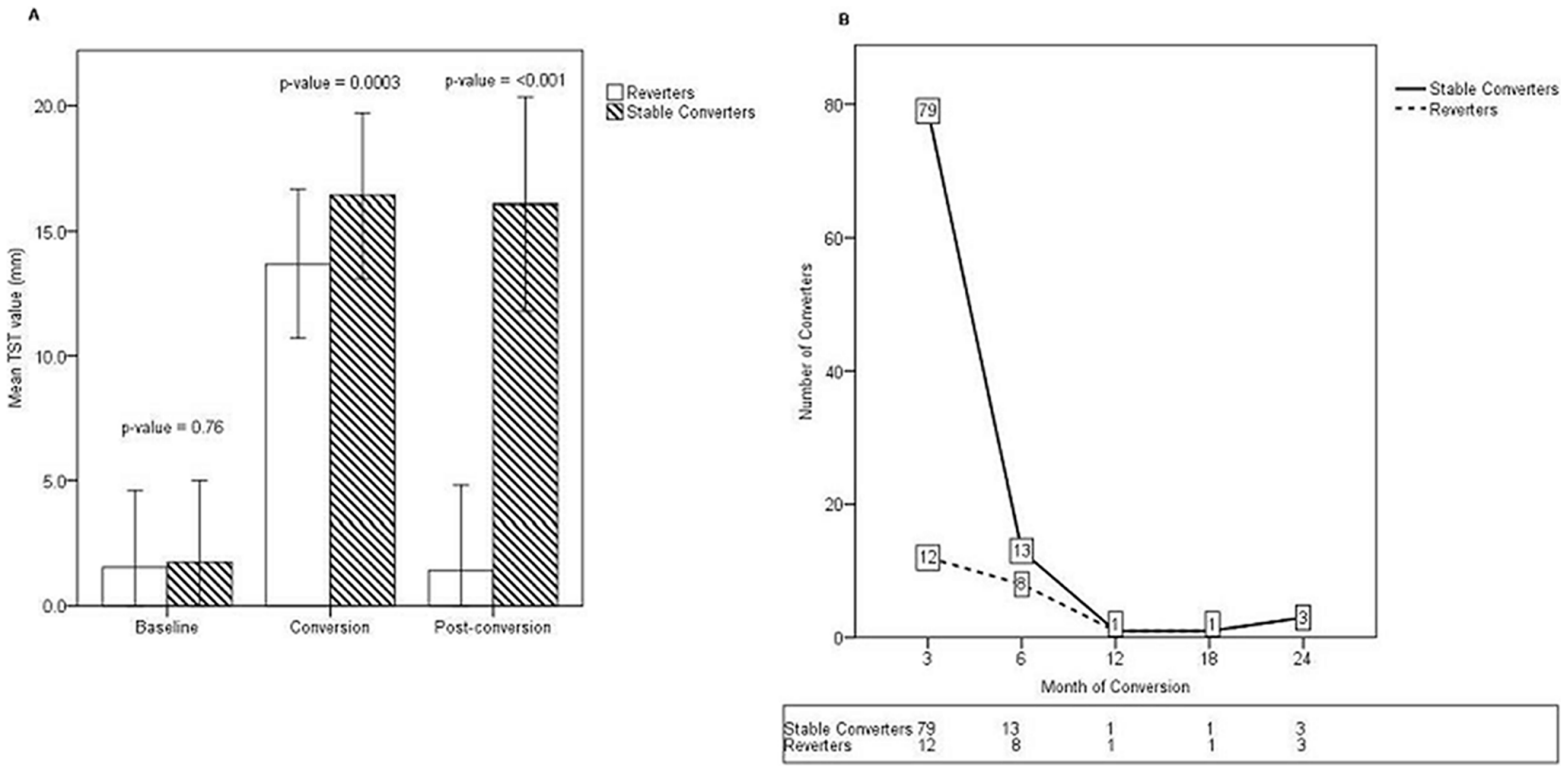
Longitudinal TST measurements among HHC. TST were placed at study enrollment and every 3-month course of INH preventive therapy and post-conversion TST was placed 12 months following each subject's initial conversion event. Following measurement of post-conversion TST, subjects were classified as either stable converters or reverters. Shown are mean TST measurements (mm) ± 1 SD for all eligible study participants at study enrollment (baseline), conversion, and post-conversion time points (A). As shown in Panel B (numbers in boxes indicate number of conversions per time point), subjects with stable conversions were more likely to experience their initial conversion soon following study entry (mean time to conversion, 4.1 months) while reverters experienced their initial conversion later (mean time to conversion 7.2 months; p = 0.03).

At study entry, all participants had negative TSTs, with mean TST measurements of 1.8 mm and 1.6 mm in stable converters and reverters, respectively (p = 0.76; Figure 1A). As shown in Figure 1A, both groups had conversion TST measurements well above a 10 mm cut-off, with stable converters having significantly larger areas of induration compared to reverters (16.4 mm *vs.* 13.7 mm, respectively; p = 0.003). The mean change in TST measurements between enrollment and conversion were also notably different between groups, with an increase of 14.6 mm noted for stable converters and a mean change of 12.1 mm for individuals who would go on to revert (p = 0.01). After completion of a 9 month course of IPT, all subjects underwent repeat TST 12 months following their conversion event (post-conversion TST). As shown in Figure 1A **,** post-conversion TST measurements remained well above 10 mm for stable converters (16.3 mm), while the mean TST measurement among reverters was significantly less (1.4 mm; p<0.001). Time to initial TST conversion was also noted to be significantly different between stable converters and reverters. As shown in Figure 1B, the majority of stable converters had their initial TST conversion event soon after study enrollment, with a mean time to conversion of 4.1 months. Reverters experienced their initial TST conversions later, with a mean time to conversion of 7.2 months following enrollment (p = 0.03).

### Serial WB-IGRA results among study participants

**Figure 2:**
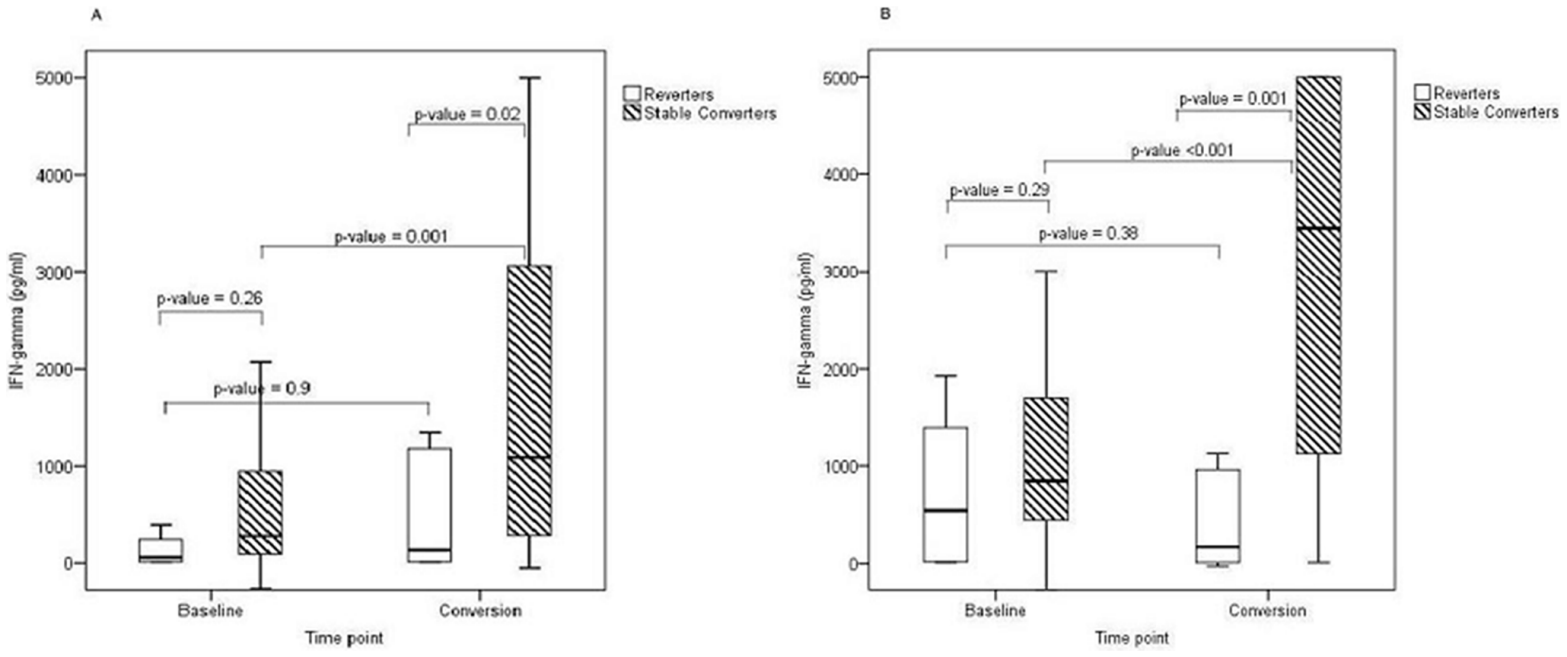
Longitudinal WB-IGRA results among HHC. Whole blood was obtained from a subset of eligible subjects at study enrollment (baseline) and time of initial TST conversion. WB-IGRA were performed using Mtb Ag85B (A) and whole Mtb H37Ra (B) as T cell stimulus. Boxes indicate the interquartile ranges, horizontal lines transecting boxes indicate medians, and whiskers indicate highest and lowest values. At study entry (baseline), no significant differences were noted between reverters and stable converters for either stimulus. At the time of conversion, subjects with stable conversion produced significantly more IFN-γ than reverters in response to both Mtb Ag85B and whole Mtb H37Ra (A, B). Moreover, IFN-γ production significantly increased among stable converters between enrollment and conversion, while there were no significant changes noted for reverters. Statistical comparison between groups was performed using Wilcoxon rank sum test, and comparison with groups performed with Wilcoxon sign-rank test (p< 0.05 considered significant).

Our in-house WB-IGRA was performed in a subset of HHC at study enrollment and at initial TST conversion (n = 6 and n = 21 for Ag85B; n = 8 and n = 34 for Mtb H37Ra; reverters and stable converters, respectively), prior to initiation of IPT. At study enrollment (baseline samples), there were no significant differences in median IFN-γ production in response to Mtb Ag85B or whole Mtb H37Ra between HHC who would go on to be stable TST converters and TST reverters (Figure 2A,B). Although all subjects were TST negative at enrollment, both groups had detectable responses to Mtb Ag85B (40.7 pg/ml and 242.7 pg/ml for reverters and stable converters, respectively, p = 0.26) and Mtb H37Ra (874.8 pg/ml and 889.1 pg/ml for reverters and stable converters, respectively, p = 0.29). When the WB-IGRA was repeated at time of TST conversion, however, significant differences were noted between groups. Among stable converters, IFN-γ responses to Ag85B significantly increased from baseline to conversion, with a median value of 1317.2 pg/ml at conversion (p = 0.001); for future TST reverters, however, there was no significant increase in IFN-γ production to Ag85B between time points (p = 0.9). Moreover, when comparing the two groups, stable converters produced significantly more IFN-γ in response to Ag85B than future reverters at time of conversion (p = 0.02). WB-IGRA results at conversion in response to Mtb H37Ra demonstrated a similar pattern as that observed with Ag85B. For stable converters, IFN-γ production increased significantly from baseline to conversion (p<0.001), while responses among future reverters did not significantly change, although a downward trend was noted (p = 0.38). In addition, at the time of TST conversion, stable converters produced significantly more IFN-γ in response to Mtb H37Ra than future reverters (p = 0.001). Median change in IFN-γ production in response to Ag85B and Mtb H37Ra from enrollment to conversion was also significantly different between groups (p = 0.02 and p = 0.002, respectively).

### Exposure risk factors of subjects with stable TST conversions and TST reversions

To be eligible for this study, all subjects were HHC contacts of an adult with smear and culture positive Pulmonary TB. However, we were interested in exploring additional epidemiologic risk factors to quantify the degree of Mtb exposure between subjects with stable TST conversions and TST reversions. As shown in Table 2, there was no significant difference in degree of sputum smear positivity among household index cases between the two groups, nor were there differences in the presence of cavitary lesions on enrollment CXRs. Moreover, there were no differences in the number of co-prevalent (defined as development of TB disease within 3 months of study enrollment) or incident (defined as development of TB disease after 3 months of study enrollment) household TB cases between subjects with stable TST conversions and TST reversions. To further refine our measure of exposure risk, we examined our modified TB exposure score specific to TB HHC originally developed by Mandalakas *et al*., [22] (see Table S1) to compare the degree of close contact with the index case among study participants. On our modified risk assessment scale, both stable converters and reverters had a median risk score of 6.0 (range 5–10 and 5–8, respectively), with no significant difference between the two groups (p = 0.74).

**Table 2:** Epidemiologic Measures of TB exposure burden.

	Totaln = 122	Stable Convertersn = 97 (79.5%)	Revertersn = 25 (20.5%)	P*-*value
AFB smear of Index				
1+	5	4 (4%)	1 (4%)	0.52
2+	25	18 (19%)	7 (28%)	
3+	92	75 (17%)	17 (68%)	
Cavitary lesions on Index CXR				
Yes	66	53 (55%)	13 (52%)	0.81
No	55	43 (44%)	12 (48%)	
Unknown	1	1 (1%)	0	
# Co-prevalent TB cases in household				
0	105	84 (87%)	21 (84%)	0.96
1	9	6 (6%)	3 (12%)	
2	8	7 (7%)	1 (4%)	
# Incident TB cases in household				
0	114	92 (95%)	22 (88%)	0.10
1	7	5 (5%)	2 (8%)	
2	1	0	1 (4%)	

## Discussion

TST reversion has been reported for decades, most commonly in the context of IPT among healthy Mtb contacts [5]–[8]; [23]–[28]. Reported TST reversion rates vary widely, and differences in study design, study populations, and ability to control for potential confounders such as timing of initial TST conversion, repeated Mtb exposures, chronic NTM exposure, and receipt of BCG vaccine have made it difficult to draw conclusions regarding the frequency and biologic significance of reversion. Specifically, whether reversion following IPT reflects successful clearance of Mtb infection, non-specific variation in tuberculin sensitivity, or declining Mtb-specific cell mediated immune responses, remains controversial [9]. Moreover, few studies have used serial TSTs combined with Mtb specific IGRAs to measure Mtb-infection status in a well-characterized cohort of individuals with recent, documented exposure. Using a prospective household contact design, we identified a group of Mtb-exposed individuals who were TST negative at enrollment with subsequent TST conversion; thus capturing acute and presumptively primary, Mtb infection. Although the majority of individuals remained persistently TST positive (“stable converters”) following IPT, a substantial proportion (20.5%) reverted their TST (“reverters”). Thus, even in a TB endemic setting in a population of BCG vaccinated and NTM exposed individuals with recent Mtb exposure, TST reversion occurred in one of five individuals prescribed IPT.

Our study confirmed a key observation regarding TST conversion and reversion reported by other groups, that individuals who go on to revert their TST have significantly smaller TSTs at conversion than stable converters. A critical component of studies of TST conversions and reversions are the clinical definitions of these events. We selected criteria shown to reduce the chance that random variations in TST measurements over time would be categorized as a conversion or reversion [20]. Although our study definitions (3 mm for <5 year old; 6 mm for >5 years old) are less strict than those currently recommended by groups such as the American Thoracic Society that define TST conversion as an increase in ≥ 10 mm induration over a one year period [18], the actual changes in TST measurements demonstrated by our study participants were within this more stringent 10 mm range for both TST conversions and reversions. Remarkably, among TST reverters, their median change in induration 12 months following conversion was a reduction by 12.1 mm. Therefore, the conversions and reversions captured by this study are unlikely to represent random variations over time, but rather biomarkers of significant immunologic responses to Mtb infection.

An alternative interpretation of our TST findings is that use of serial TST measurements resulted in a boosting phenomenon among some participants; thus, their TST conversion reflects recollection of pre-existing immune sensitization to mycobacterial antigens or BCG vaccination, rather than primary Mtb infection. Baseline two-step TST testing has been proposed as a technique to distinguish boosting from genuine TST conversion [20]. However, two-step TST testing was not incorporated into our study design; rather participants received serial TSTs placed in 90–180 day intervals. Despite this limitation, we propose that the TST conversions observed in this study reflect genuine Mtb infection as: 1) all participants had significant exposure to confirmed cases of smear-positive pulmonary TB prior to conversion; 2) TST conversions were large in absolute size; 3) boosting is less common when repeat TST is performed 90+ days from initial testing [20], [29].

An unexpected finding of our study was that time to initial TST conversion was significantly different between stable converters and reverters. In fact, those who would go on to revert their TST experienced initial conversion 7.2 months following enrollment, while stable converters developed their positive TST at 4.1 months. Although we hypothesized that reverters had a reduced burden of Mtb exposure, thus explaining their delayed TST conversion, exposure risks (smear positivity of index case, cavities on chest x-rays of index case, presence of multiple TB cases in the household) were not different between reverters and stable converters. Therefore, we propose that a unique host response to Mtb-infection, rather than differences in the burden of exposure, is responsible for both TST reversions and stable IGRA responses observed in our reverter group.

Although there were no significant demographic, clinical, or epidemiologic differences between TST reverters and stable converters, these groups demonstrated immunologic divergence beyond that reflected by serial TST measurements. Firstly, at study enrollment there were no significant differences between groups in Mtb-specific T cell IFN-γ production as measured by our 7 day WB-IGRA. In fact, although all subjects were, by definition, TST-negative at enrollment, both groups already had evidence of Mtb-sensitization by WB-IGRA. This observation re-enforces early reports that a detectable delayed-type sensitivity reaction resulting in a positive TST forms 2–10 weeks following primary infection with Mtb [30], and contributes to an emerging literature exploring time to IGRA conversion following known Mtb exposure [31]. Notably, we observed that at initial TST conversion, those who would go on to revert their TST following INH treatment had no significant changes in Mtb-specific T cell IFN-γ responses compared to their enrollment, pre-TST conversion, values. This is in contrast to stable converters who demonstrated significant increases in WB-IGRA responses from enrollment to conversion. Thus, even prior to initiation of IPT, a sub-set of individuals experiencing acute Mtb-sensitization as detected by TST conversion, demonstrate a pattern of Mtb-specific T cell responses predictive of future TST reversion. Although our study could not directly measure burden of Mtb-infection in participants, we hypothesize that reverters have unique early immune responses to Mtb-infection that minimize and perhaps even eliminate bacillary growth and results in: 1) delayed TST conversion with less induration, and 2) stable T cell effector responses to Mtb prior to provision of IPT.

Several groups have reported change in IGRA responses among healthy, Mtb-exposed, TST-positive individuals both with and without preventive anti-mycobacterial regimens [12]; [32]–[35]. Similar to earlier reports of TST reversion, these studies have found that declining or “reverted” IGRA results are more common in individuals who received preventive therapy and those recently exposed. The majority of studies have used commercial IGRAs (T-SPOT.TB or Quantiferon Gold) or other short-term in-house assays with ESAT-6 and CFP-10 peptide pools. It remains unclear if the observed diminished frequency of Mtb-specific T cells is due to successful eradication or reduction of Mtb burden, or an artifact of the antigens most commonly employed in these assays, ESAT-6 and CFP-10. Namely, it is possible that to maintain persistently positive IGRA responses to Mtb using short term assays, effector T cells must enjoy continued *in vivo* exposure to tested antigens. If Mtb enters a latent state following acute infection, it may no longer secrete ESAT-6 and CFP-10 and thus IGRA responses against these antigens would decline [35], [36]. Our study differed as we used whole avirulent Mtb bacilli and Antigen 85B protein to elicit an Mtb-specific T cell response. In order to allow time for antigen processing and presentation, and to stimulate both effector and central memory T cells, our cell cultures were incubated for 7 days as opposed to the short term, overnight incubations utilized in commercial IGRAs. In fact, as other investigators have shown non-specific 6 day IGRA responses to Mtb sonicate and culture filtrate among BCG-vaccinated individuals without known Mtb-exposure [37], it is striking that we detected significant differences between our two groups using whole Mtb. Not only were there robust differences in IFN-γ production between stable-converters and reverters at TST conversion, but these groups demonstrated distinct patterns of IFN-γ production from study enrollment to conversion with stable converters experiencing an increase in IFN-γ production while reverters maintained their pre-conversion responses.

Our study has several limitations to be discussed. First, we had limited specimens available to perform our WB-IGRA assay and this prevented longitudinal analysis of individual WB-IGRA results. Because the study was not originally designed to identify reverters and analyze their immunological responses, it was not powered to observe statistically significant differences. Using cross-sectional analysis we were able to show clear differences in median change of IFN-γ production from study enrollment to conversion between stable converters and reverters. It is possible, however, that our conclusion that WB-IGRA results were equivocal at study enrollment between reverters and stable converters reflects a Type II error due to inadequate power. We do not have specimens available from post-IPT time points for WB-IGRA; therefore we cannot assess if the divergent IGRA results observed between groups at TST conversion were maintained following IPT, nor if WB-IGRA responses in reverters were reduced to undetectable. We also noted that among the 219 healthy, HIV-uninfected, TST-negative HHC who converted during the study and accepted IPT, 97 could not be included in this sub-study as they were missing post-conversion TST measurements. Therefore, we cannot say with certainly that our results are reflective of the entire study population of TST converters, as 44% could not be included in our analysis. As discussed previously, adherence to IPT was not formally assessed in this study. Therefore, we cannot determine if TST reversion was more or less common among individuals who were fully compliant with IPT versus those non-compliant with IPT. Finally, as participants who remained healthy were not repeatedly tested for HIV-infection, it is possible that the incidence of acute HIV infection differed between reverters and stable converters. However, given the young age of our participants (72% of reverters and 63% of stable converters under 15 years), it is unlikely that un-recognized acute HIV infection substantially impacted our results.

We submit that within our group of recently exposed individuals with primary Mtb-immune sensitization, the combined findings of stable WB-IGRA responses with subsequent TST reversion, identify a sub-population of Mtb-infected individuals with a unique host response to Mtb. Future studies of Mtb-infection should focus on elucidating if this unique host immunologic phenotype reflects successful reduction in bacillary burden and thus serves as a correlate of protective immunity against development of TB. Further development of refined assays that establish patterns of IFN-γ responses among Mtb-exposed individuals and that distinguish individuals with persistent infection from those who have eliminated Mtb will significantly advance TB-control efforts and targeted preventive therapy programs in resource limiting settings.

## Supporting Information

Table S1 Modified Risk Assessment Score for Close Contact with Index Case.
(DOCX)
