# Supplementary material for: Tuberculin Skin Test Reversion following Isoniazid Preventive Therapy Reflects Diversity of Immune Response to Primary Mycobacterium tuberculosis Infection
Source: PLoS One. 2014 May 5;9(5):e96613. doi: 10.1371/journal.pone.0096613 (PMC4010490; doi:10.1371/journal.pone.0096613)
Supplement: Table S1 — Modified Risk Assessment Score for Close Contact with Index Case. (DOCX) [file pone.0096613.s001.docx]

**Supplemental Table 1: Modified Risk Assessment Score for Close Contact with Index Case***

| Index is AFB smear positive |
| --- |
| Index has pulmonary TB |
| Index with active cough |
| Index lives in household |
| Index is Mother (child) or Spouse (adult) |
| Sleep in same room as Index |
| Sleep in same bed as Index |
| Daily contact with Index |
| Multiple TB contacts in home |

*modified from Mandalakas, et al [22].
